# Analysis of readability and structural accuracy in SNOMED CT

**DOI:** 10.1186/s12911-020-01291-y

**Published:** 2020-12-15

**Authors:** Francisco Abad-Navarro, Manuel Quesada-Martínez, Astrid Duque-Ramos, Jesualdo Tomás Fernández-Breis

**Affiliations:** 1 Departamento de Informática y Sistemas, Universidad de Murcia, Campus de Espinardo, 30100 Murcia, Spain; 2 Instituto Murciano de Investigación Biosanitaria (IMIB-Arrixaca), Hospital Clínico Universitario Virgen de la Arrixaca, 30120 Murcia, Spain; 3 Center of Operations Research (CIO), Miguel Hernández University of Elche, Avda. de la Universidad, 03202 Alicante, Spain; 4 Facultad de Ingenierías, Universidad Autónoma Latinoamericana, Carrera 55 49, 050010 Medellín, Colombia

**Keywords:** Ontologies, Quality assurance, Quality metrics, Readability, Structural accuracy

## Abstract

**Background:**

The increasing adoption of ontologies in biomedical research and the growing number of ontologies available have made it necessary to assure the quality of these resources. Most of the well-established ontologies, such as the Gene Ontology or SNOMED CT, have their own quality assurance processes. These have demonstrated their usefulness for the maintenance of the resources but are unable to detect all of the modelling flaws in the ontologies. Consequently, the development of efficient and effective quality assurance methods is needed.

**Methods:**

Here, we propose a series of quantitative metrics based on the processing of the lexical regularities existing in the content of the ontology, to analyse readability and structural accuracy. The readability metrics account for the ratio of labels, descriptions, and synonyms associated with the ontology entities. The structural accuracy metrics evaluate how two ontology modelling best practices are followed: (1) lexically suggest locally define (LSLD), that is, if what is expressed in natural language for humans is available as logical axioms for machines; and (2) systematic naming, which accounts for the amount of label content of the classes in a given taxonomy shared.

**Results:**

We applied the metrics to different versions of SNOMED CT. Both readability and structural accuracy metrics remained stable in time but could capture some changes in the modelling decisions in SNOMED CT. The value of the LSLD metric increased from 0.27 to 0.31, and the value of the systematic naming metric was around 0.17. We analysed the readability and structural accuracy in the SNOMED CT July 2019 release. The results showed that the fulfilment of the structural accuracy criteria varied among the SNOMED CT hierarchies. The value of the metrics for the hierarchies was in the range of 0–0.92 (LSLD) and 0.08–1 (systematic naming). We also identified the cases that did not meet the best practices.

**Conclusions:**

We generated useful information about the engineering of the ontology, making the following contributions: (1) a set of readability metrics, (2) the use of lexical regularities to define structural accuracy metrics, and (3) the generation of quality assurance information for SNOMED CT.

## Background

Recently, ontologies and terminologies, such as the SNOMED CT [1], the Gene Ontology (GO) [2, 3], or the Disease Ontology (DO) [4], have demonstrated their usefulness for supporting biomedical research, thus making their quality and maintenance critical. In general, ontologies have been defined as ‘a formal, explicit specification of a shared conceptualization’ [5], and their use in biomedicine is mostly because ontologies facilitate knowledge and data sharing and interoperability. For instance, GO is used in biological sciences for predicting gene functions [6] or protein locations [7–9], whereas SNOMED CT is a key component to enable interoperability across different healthcare systems [10–13]. Ontologies must be friendly for both humans and machines. Practically speaking, the human-oriented content is expressed in natural language and the machine-oriented one as axioms.

In general, the quality assurance process ensures that the design requirements are met. Hence, this process must include methods for identifying flaws and, ideally, for proposing corrective actions. When approaching the ontology quality evaluation or quality assurance, the first problem is the lack of a standardised view in the community about these processes. In the literature, the measurement and analysis of characteristics, such as consistency, coverage, completeness, structural accuracy, functional adequacy, or readability, among others, have been identified as relevant [14–17]. Once the characteristics have been identified, the second aspect is to define the qualitative or quantitative metrics to measure them. A variety of metrics dealing specially with the structural aspects of the ontology have been proposed in the last few years. For example, the metrics ‘lack of cohesion in methods’ [18], ‘tangledness’ [15], ‘semantic variance’ [19], or those related to ‘ontology richness’ defined in [20, 21] use the semantic information stored in the ontology for quantifying structural aspects. Other examples are the metrics defined in [22, 23], which use an extra corpora of domain-related documents for measuring the coverage of ontologies in a specialised domain, or metrics based on semiotics [24].

Given that the content of the ontology has to be friendly for both humans and machines, our hypothesis in this work is that the analysis of the correspondence between the content for humans and machines should provide relevant information for the quality assurance of the ontology. Therefore, in this study, we addressed two specific ontology features, namely, readability and structural accuracy. The first one was defined as the existence of human readable descriptions in the ontology, such as comments, labels, or captions [20]; thus, it is related to the amount of content in natural language for humans, whereas the second one is defined as the ability to appropriately represent semantics as understood by society [19], hence being related to the content for the machines.

Readability has been traditionally measured in terms of the average number of names, synonyms, and descriptions per ontology entity, namely classes and properties. Here, we propose metrics based on the annotation properties that are mostly used by the semantic web community. This will be done by analysing the content of BioPortal ontologies. In contrast, structural accuracy has been mostly evaluated by domain experts. We propose to process the content in the natural language as a way for increasing the automation of this evaluation, which will simplify the quality assurance processes. For this purpose, we will exploit the notion of ‘hidden semantics’ [25], which states that a considerable amount of the semantics of the ontology entities is encoded in the content in the natural language but not in the axioms. Concretely, we propose metrics that will exploit the content of the class labels, which have been presumably written by domain experts. We propose two metrics related to the structural accuracy: (1) systematic naming, which measures the lexical similarity between the labels of classes and their descendant classes; and (2) lexically suggest logically define, which measures how aligned the label and the axioms of a class are, as this principle implies that what is expressed in the natural language for humans should be available for the machines as axioms.

We will illustrate the usefulness of these metrics for ontology quality assurance by evaluating all of the SNOMED CT versions from 2011 to 2019. We will pay special attention to the most recent version available at the time of this study. The readability metrics will provide a quantitative description of the content in the natural language. The structural accuracy metrics not only provide a quantitative description of the structural aspects of the ontology but also permit to detect the possible missing semantic relations in the ontology, in cases in which a lexical relation between classes is not logically formulated as an axiom. We selected SNOMED CT for this study because of its relevance in health informatics, its maturity, and its continuous evolution by SNOMED International.

We believe that our work will contribute by providing readability metrics based on the most frequently used annotation properties, and structural accuracy metrics based on the analysis of both the lexical and the logical content of the ontology by the study of lexical regularities among the ontology classes. We believe that these metrics can help to make quality assurance processes more efficient and effective. Moreover, they can help ontology content editors to make informed decisions.

The rest of the article is structured as follows. First, we will discuss some state-of-the-art solutions in the field of ontology quality assurance. Then, in the ‘Methods’ section, we will propose different metrics covering readability and structural accuracy. In the ‘Results’ section, these metrics are applied to different SNOMED CT versions from 2011 to 2019. These results are discussed in the ‘Discussion’ section. Finally, some conclusions are presented in the last section.

## State of the art

The ontology engineering community has proposed different features that should be measured for assessing the quality of ontologies. Gangemi et al. [16] proposed three main types of measures for evaluation: structural measures that are typical of ontologies represented as graphs; functional measures that are related to the intended use of an ontology and its components; and usability-profiling measures that depend on the level of annotation of the considered ontology. Rogers [17] identified four broad properties in ontologies that may be quality assured: philosophical validity, compliance with meta-ontological commitments, content correctness, and fitness for purpose. For example, Cui et al. [14] stated that ontology quality assurance attempts to assess and improve the overall quality of ontologies in the following aspects: (1) consistency of the ontological structure with respect to the explicit and implicit knowledge that they capture; (2) coverage and completeness of the ontology in terms of the classes and the properties needed to support specific applications; (3) non-redundancy of classes and properties; and (4) clarity of class and relationship definitions to enable accurate machine readability and human interpretation. In Lantow [26], three dimensions for ontology quality were proposed: domain scope, which refers to how well the ontology represents the real world; conceptual scope, which assesses the quality of the ontology in terms of software quality characteristics; and application scope, which evaluates how useful the ontology is as a component of a larger system. Our research group has contributed to this area of knowledge with OQuaRE [15], which provides an ontology quality model based on SQuaRE [27], which is a standard quality model used for software quality. The OQuaRE quality model proposes the following characteristics: structural, functional adequacy, reliability, efficiency, operability, compatibility, maintainability, transferability, and quality in use.

The community has proposed quantitative approaches based on metrics, which provide information about the engineering of the ontology [15, 18, 20, 21, 24, 28–34]. For example, Yao et al. [18], Tartir and Arpinar [20, 21], and Lantow and Birger [26] defined a series of metrics for evaluating the structural properties in the ontology. Works such as [28, 29, 35] evaluate the ontology from a realism-based perspective that demands the manual judgement of users.

In this work, we are interested in measuring readability, which has been targeted by the community in the past. Protégé [36], which is one of the most used ontology editors, provides the basic metrics related to readability, such as the annotation property count and the number of annotation assertions in the ontology. Other works use these counts to calculate a metric over the number of classes, such as OQuaRE [15], which defines the annotation richness as the mean number of annotations per class. Nonetheless, annotation properties can be used for representing elements that are not directly related to the readability, such as version information (owl:priorVersion, owl:versionInfo), deprecation information (owl:deprecated), or source definition information (rdfs:isDefinedBy). In this sense, projects such as OntoQA [20] or OntoMetrics [26] measure the readability for a class by counting the number of annotations of the types rdfs:label and rdfs:comment that the class has. Nonetheless, there exist other well-known annotation properties related to readability that are not taken into account. For example, Simple Knowledge Organization System (skos) [37] or Dublin Core Terms (dcterms) [38] provides readability-related annotation properties, such as skos:prefLabel, for setting a preferred label for a concept, or dc:description, for describing concepts.

Different methods have been proposed for measuring the structural accuracy of ontologies, which can be classified as semantic-based or lexical-based approaches. An example of a semantic-based method is presented in [39], where the authors identify structural ontology characteristics which are usually observed in high-quality ontologies. They used twelve different topological metrics and concluded that the ontologies that presented higher values of depth and breadth variance could provide better semantic relations, although the results did not reach statistical significance. On the basis of this work, the authors of [19] introduced the concept of semantic variance, which is a mathematically coherent extension of the standard numerical variance to measure the semantic dispersion of the taxonomic structure of ontologies. This semantic variance is defined as the average of the squared semantic distance between each ontology class to the ontology root. This measure was proposed as a good predictor of the ontological accuracy. In contrast, other works rely on the lexical part of the ontologies, aiming to discover new semantic definitions taking into account the hints that the lexicon provides [25]. Although we found useful works for enriching and correcting ontologies by using this approach, we did not find metrics for measuring this aspect. For example:

- In [40], the authors introduced the ‘lexically suggest, logically define’ principle, which states that the lexical content should also be represented as logical axioms. This principle was applied in [41] to suggest logical axioms based on an analysis of the lexical content.
- In [42–44], the authors grouped concepts according to a lexical similarity measure, forming similarity sets, whose members had at least five words, and they only differed from the other members at the most in one word. Then, they analysed the semantics of each similarity set in order to propose hierarchical, attribute related, or role-group related inconsistencies.
- In [45], the authors introduced a structural-lexical approach for auditing SNOMED CT by using a combination of non-lattice subgraphs of the underlying hierarchical relations and enriched lexical attributes of fully specified concept names. In this approach, new is-a relationships were suggested by means of the study of the lexicon along with the concept hierarchy.

## Methods

This section describes the metrics that we developed for measuring readability and structural accuracy, as well as the method we propose to evaluate their application to the quality assurance of a given ontology. The metrics were implemented in our OntoEnrich framework [46], which offers a number of ontology quality assurance tasks based on the lexical analysis of ontologies.

### Lexical regularities

The structural accuracy metrics defined in this work make use of lexical regularities (LR) [46], which are text patterns that appear recurrently along the class labels. In order to detect the LRs, we processed the rdfs:label annotations of the classes. In the case of SNOMED CT, these labels were derived from the *Fully Specified Name* (FSN), and we removed the semantic category from them in order to avoid the appearance of the extra LRs. Then, we used the OntoEnrich NLP algorithms to extract the LRs. OntoEnrich was executed with the following configuration: the blank character as the token delimiter, case insensitive strategy, and a coverage of 0.1, which implied that only patterns that appeared in the label of at least 10% of the ontology classes were considered LRs. When the full label of a class corresponded to an LR found in the ontology, we called this class an LR class. For example, *oral* was an LR in SNOMED CT, as it was exhibited in the labels of many classes. Moreover, it was an LR class, as there was a class in SNOMED CT whose full label was ‘oral’.

**Table 1:** Top 50-most frequently used annotation properties in BioPortal

Annotation property	Usage per entity
http://www.w3.org/2004/02/skos/core#prefLabel	0.52
http://bioportal.bioontology.org/ontologies/umls/tui	0.44
http://www.w3.org/2000/01/rdf-schema#label	0.42
http://bioportal.bioontology.org/ontologies/umls/cui	0.42
http://www.geneontology.org/formats/oboInOwl#hasDbXref	0.37
http://www.w3.org/2004/02/skos/core#altLabel	0.35
http://www.geneontology.org/formats/oboInOwl#id	0.16
http://www.geneontology.org/formats/oboInOwl#hasExactSynonym	0.10
http://www.geneontology.org/formats/oboInOwl#hasOBONamespace	0.10
http://www.geneontology.org/formats/oboInOwl#hasRelatedSynonym	0.08
http://www.w3.org/2004/02/skos/core#relatedMatch	0.08
http://www.w3.org/2004/02/skos/core#exactMatch	0.08
http://purl.obolibrary.org/obo/IAO_0000115	0.08
http://ncicb.nci.nih.gov/xml/owl/EVS/Thesaurus.owl#P383	0.07
http://ncicb.nci.nih.gov/xml/owl/EVS/Thesaurus.owl#P90	0.07
http://ncicb.nci.nih.gov/xml/owl/EVS/Thesaurus.owl#P384	0.07
http://www.w3.org/2004/02/skos/core#definition	0.07
http://www.geneontology.org/formats/oboInOwl#inSubset	0.04
http://www.w3.org/2000/01/rdf-schema#comment	0.04
http://purl.obolibrary.org/obo/IAO_0000117	0.03
http://www.loc.gov/mads/rdf/v1#hasNarrowerAuthority	0.03
http://ncicb.nci.nih.gov/xml/owl/EVS/Thesaurus.owl#A8	0.03
http://www.w3.org/2000/01/rdf-schema#seeAlso	0.03
http://purl.obolibrary.org/obo/IAO_0000119	0.03
http://purl.org/dc/terms/identifier	0.03
http://purl.obolibrary.org/obo/IAO_0000118	0.03
http://schema.org/name	0.03
http://www.w3.org/2004/02/skos/core#inScheme	0.03
http://www.geneontology.org/formats/oboInOwl#source	0.03
http://schema.org/sameAs	0.02
http://purl.org/sig/ont/fma/authority	0.02
http://purl.org/sig/ont/fma/author	0.02
http://ncicb.nci.nih.gov/xml/owl/EVS/Thesaurus.owl#P378	0.02
http://www.w3.org/2004/02/skos/core#broader	0.02
http://ncicb.nci.nih.gov/xml/owl/EVS/Thesaurus.owl#P106	0.02
http://purl.org/sig/ont/fma/Date_entered_modified	0.02
http://purl.org/dc/elements/1.1/description	0.02
http://ncicb.nci.nih.gov/xml/owl/EVS/Thesaurus.owl#NHC0	0.02
http://ncicb.nci.nih.gov/xml/owl/EVS/Thesaurus.owl#P108	0.02
http://www.geneontology.org/formats/oboInOwl#hasSynonymType	0.02
http://purl.obolibrary.org/obo/OGG_0000000015	0.02
http://purl.obolibrary.org/obo/OGG_0000000018	0.02
http://purl.obolibrary.org/obo/OGG_0000000006	0.02
http://purl.obolibrary.org/obo/OGG_0000000019	0.02
http://purl.obolibrary.org/obo/OGG_0000000009	0.02
http://purl.obolibrary.org/obo/OGG_0000000017	0.02
http://ncicb.nci.nih.gov/xml/owl/EVS/Thesaurus.owl#P97	0.02
http://purl.org/dc/terms/subject	0.02
http://www.geneontology.org/formats/oboInOwl#created_by	0.02
http://ncicb.nci.nih.gov/xml/owl/EVS/Thesaurus.owl#P207	0.01

### Readability metrics

Readability has usually been measured in terms of the number of names, synonyms, and descriptions per entity (classes and properties) in the ontology. These metrics measure the human-friendly content of the ontology. In OWL ontologies, such content is defined in the annotation properties. Ontology developers can create their own annotation properties in OWL2. We inspected the use of annotation properties in BioPortal [47]. For each annotation property, we obtained their global usage ratio per entity in Bioportal, which was calculated by taking the number of uses of the annotation property in the repository and dividing it by the total number of entities. Table 1 shows the 50 most used annotation properties. Finally, we selected the annotation properties associated with names, synonyms, and descriptions (see Table 2).

**Table 2:** Identified annotations properties for describing labels, synonyms, and descriptions

Name	http://www.w3.org/2004/02/skos/core#prefLabel
	http://www.w3.org/2000/01/rdf-schema#label
	http://schema.org/name
	http://ncicb.nci.nih.gov/xml/owl/EVS/Thesaurus.owl#P108
Synonym	http://www.w3.org/2004/02/skos/core#altLabel
	http://www.geneontology.org/formats/oboInOwl#hasExactSynonym
	http://www.geneontology.org/formats/oboInOwl#hasRelatedSynonym
	http://www.geneontology.org/formats/oboInOwl#hasBroadSynonym
	http://www.geneontology.org/formats/oboInOwl#hasNarrowSynonym
	http://ncicb.nci.nih.gov/xml/owl/EVS/Thesaurus.owl#P90
	http://purl.obolibrary.org/obo/IAO_0000118
Description	http://purl.obolibrary.org/obo/IAO_0000115
	http://www.w3.org/2004/02/skos/core#definition
	http://www.w3.org/2000/01/rdf-schema#comment
	http://purl.org/dc/elements/1.1/description
	http://ncicb.nci.nih.gov/xml/owl/EVS/Thesaurus.owl#P97

Hence, our readability metrics were defined and calculated using these annotation properties:

- *Names per class*: ratio of the number of names associated with classes to the total number of classes.
- *Names per object property*: ratio of the number of names associated with object properties to the total number of object properties.
- *Names per datatype property*: ratio of the number of names associated with datatype properties to the total number of datatype properties.
- *Names per annotation property*: ratio of the number of names associated with annotation properties to the total number of annotation properties.
- *Synonyms per class*: ratio of the number of synonyms associated with classes to the total number of classes.
- *Synonyms per object property*: ratio of the number of synonyms associated with object properties to the total number of object properties.
- *Synonyms per datatype property*: ratio of the number of synonyms associated with datatype properties to the total number of datatype properties.
- *Synonyms per annotation property*: ratio of the number of synonyms associated with annotation properties to the total number of annotation properties.
- *Descriptions per class*: ratio of the number of descriptions associated with classes to the total number of classes.
- *Descriptions per object property*: ratio of the number of descriptions associated with object properties to the total number of object properties.
- *Descriptions per datatype property*: ratio of the number of synonyms associated with datatype properties to the total number of datatype properties.
- *Descriptions per annotation property*: ratio of the number of synonyms associated with annotation properties to the total number of annotation properties.

**Fig. 1:**
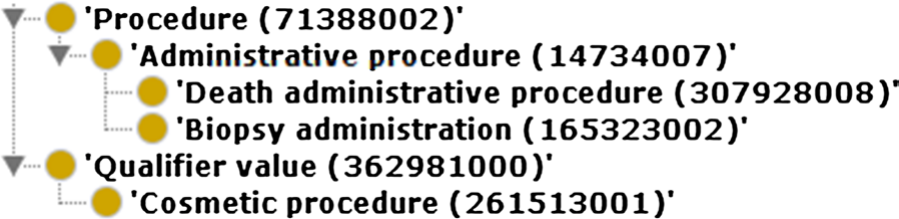
Example of hierarchy formed by concepts in SNOMED CT

**Fig. 2:**
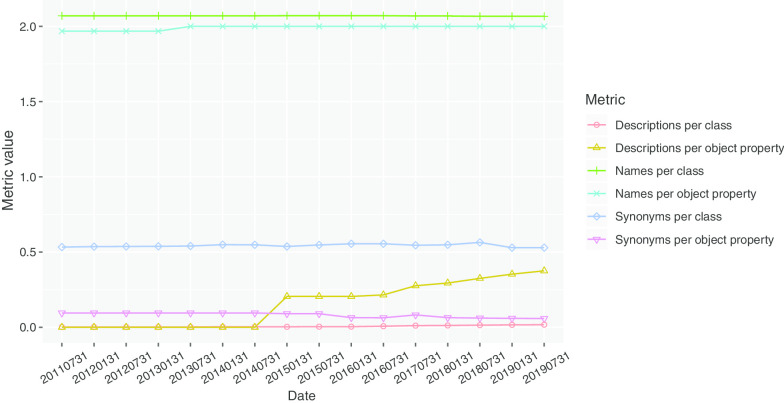
Values of the readability metrics for all of the SNOMED CT versions included in the study: 2011–2019

**Fig. 3:**
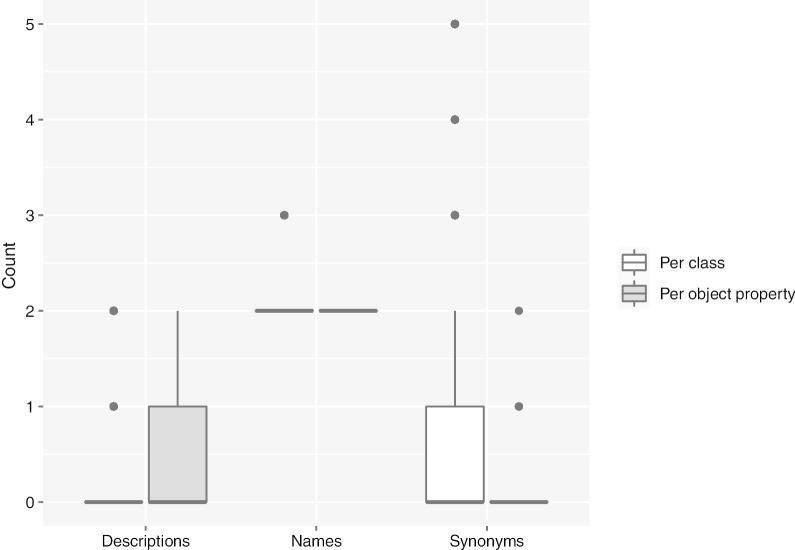
Box plots indicating the number of descriptions, names, and synonyms per class and per object property. Y axis was limited to 5 because of readability reasons

### Structural accuracy metrics

We propose to analyse the accuracy of the structure by exploiting and comparing the lexical and the logical content of the ontology. Our structural accuracy metrics will measure to what extent the lexical and logical content are aligned.

**Table 3:** Top ten LR classes according to the systematic naming metric, sorted by positive cases

LR class (label)	LR class depth	Positive cases (depth) (distance)	Negative cases (depth) (distance)	Metric value
411123000 (diagnostic allergen extract)	4	336 (5.961) (1.961)	0 (NA) (NA)	1
256259004 (pollen)	7	289 (8.606) (2.346)	0 (NA) (NA)	1
24851008 (deoxyribonucleic acid)	8	243 (10.465) (2.465)	0 (NA) (NA)	1
263490005 (status)	6	38 (7.658) (1.658)	0 (NA) (NA)	1
257351008 (shunt)	7	22 (8) (1)	0 (NA) (NA)	1
87612001 (blood)	5	20 (6.2) (1.2)	0 (NA) (NA)	1
449872003 (powder)	5	19 (6.158) (1.158)	0 (NA) (NA)	1
264193005 (segment)	7	10 (8.4) (1.4)	0 (NA) (NA)	1
255711007 (pattern)	6	9 (7) (1)	0 (NA) (NA)	1
277536004 (serogroup)	6	8 (7) (1)	0 (NA) (NA)	1

In this work, we propose two structural accuracy metrics, whose purpose is to measure the fulfilment of two of the best practices in ontology engineering:

- Lexically suggest logically define (LSLD). This principle establishes that what is expressed in a human-friendly way in the natural language should also be available as logical axioms for the machine [40]. Let us consider the example in Fig. 1, which contains a simplified hierarchy taken from SNOMED CT, showing the label together with the concept id within parentheses. In this case, the concept Procedure is an LR class because its label is contained in the labels of the classes Administrative procedure, Death administrative procedure, and Cosmetic procedure. The LSLD principle would suggest that as these classes are lexically related to Procedure, they should also be semantically related; however, Cosmetic procedure is not semantically linked with Procedure, so the lexical and logical content will not be aligned.
- Systematic naming. This principle establishes that the labels of the taxonomically related classes should share a lexical regularity. In the example shown in Fig. 1, the class labelled ‘Biopsy administration’ belongs to a hierarchy that presents some type of lexical regularity: the sibling of Biopsy administration is Death administrative procedure, which contains the label of their parent class Administrative procedure, and this class contains the label of its parent, Procedure. All of the concepts in this hierarchy, except for Biopsy administration, exhibit the LR of the parent class. This indicates that the class Biopsy administration should not be in this position of the hierarchy. Note that if it were in the correct position, it is possible that its label would not have been sufficiently descriptive.

**Fig. 4:**
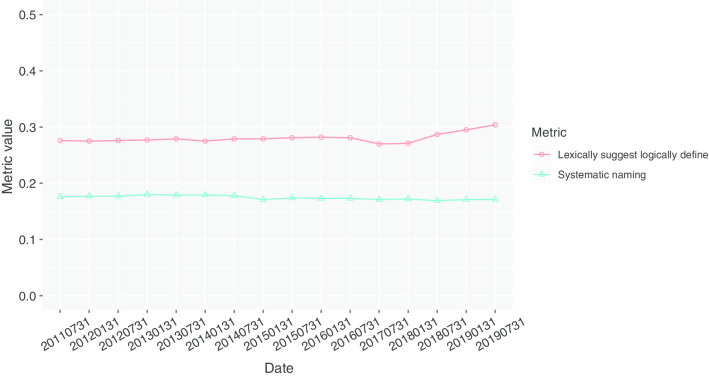
Values of the structural metrics for all of the SNOMED CT versions included in the study: 2011–2019

**Fig. 5:**
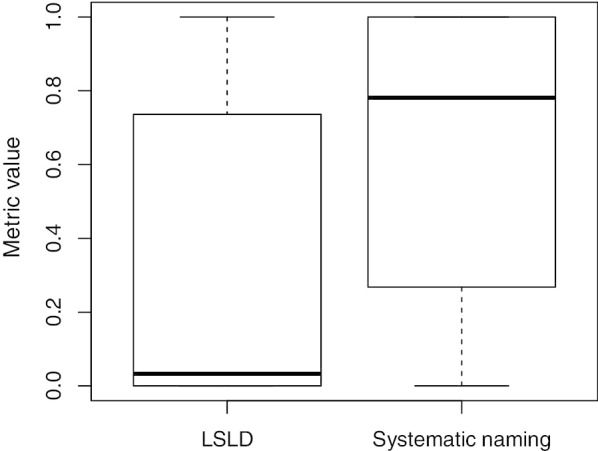
Boxplots for the LSLD and systematic naming metrics for each LR class in the SNOMED CT July 2019 release

**Fig. 6:**
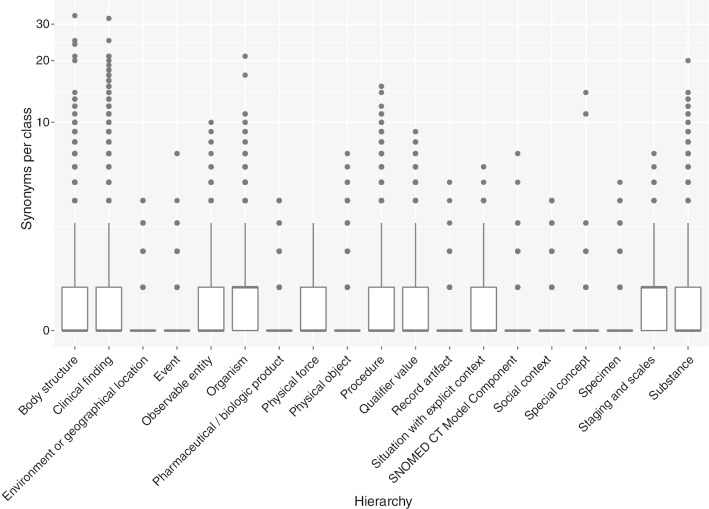
Boxplots for number of synonyms per class (log scale) by hierarchy in the SNOMED CT July 2019 release

**Fig. 7:**
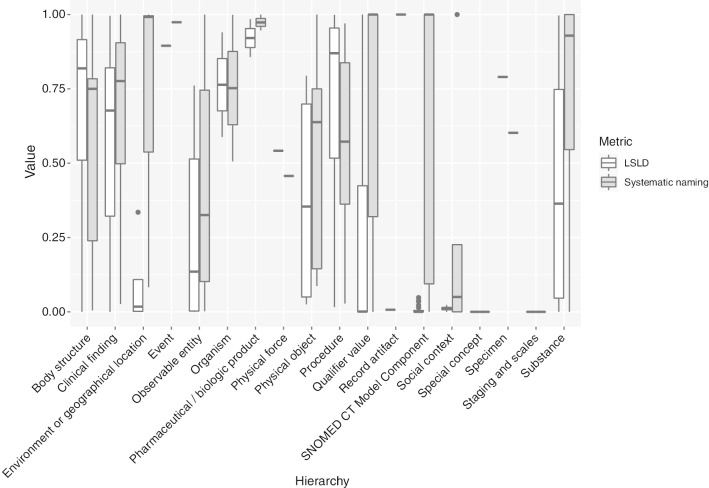
Boxplots for the distribution of both LSLD and systematic naming metrics per LR class by hierarchy in the SNOMED CT July 2019 release

#### Lexically suggest logically define (LSLD) metric

The LSLD metric, which is inspired by [40], accounts for the ratio of classes that exhibit the same LR and are logically connected in the ontology through an object property or a taxonomic link. For this purpose, only the LR associated with the LR classes is considered. It is calculated as described in Algorithm 1. In brief, we processed the labels of the ontology classes to obtain the set of lexical regularities (LRs) and the associated LR classes. Then, for each class exhibiting an LR, we checked whether there was a link between the class and the corresponding LR class; this class was classified as a positive/negative case depending on the existence of such a link. We applied the breadth-first algorithm to find the path between the classes. In particular, given a class *X*, we considered its *owl:subclassOf* and *owl:equivalentClass* axioms as edges, and the classes appearing in these axioms were the classes adjacent to *X*. Then, given this ontology graph model, the classes $C_1$ and $C_2$ were semantically related if there existed a path from $C_1$ to $C_2$ or vice versa. Note that the *owl:subclassOf* and *owl:equivalentClass* axioms were related not only to taxonomic relations but also to non-hierarchical relations through properties. Let us consider that the class ‘Structure of left upper limb’ was declared equivalent to (‘Structure of left half of body’ and hasLaterality some ‘Left’), and ‘Left’ is an LR class. Then, our algorithm would find a non-hierarchical relation between the classes ‘Left’ and ‘Structure of left upper limb ’ through the attribute hasLaterality. Therefore, ‘Structure of left upper limb’ would be detected as a positive case of the LR class ‘Left’ because it exhibited the LR ‘Left’. Finally, the ratio of positive/negative cases could be obtained. 
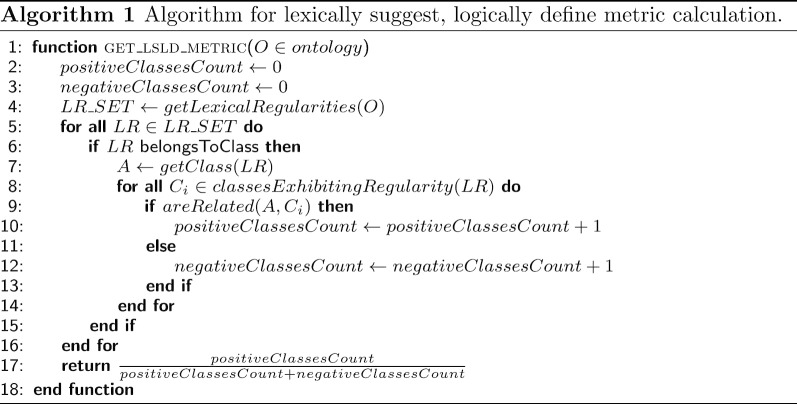


The example shown in Fig. 1 has two LR classes: Procedure and Administrative procedure. Then, for each of these LR classes, we obtained the classes whose labels exhibited its lexical regularity. On the one hand, the classes Death administrative procedure, Administrative procedure, and Cosmetic procedure exhibited the LR ‘Procedure’. The classes Death administrative procedure and Administrative procedure were semantically linked to Procedure, but Cosmetic procedure was not. On the other hand, the LR ‘Administrative procedure’ was only exhibited by the class Death administrative procedure, in addition of the corresponding LR class. In this case, the classes Administrative procedure and Death administrative procedure were semantically related. Then, the value of the LSLD metric for this example was 3/4, as there were three positive cases and one negative case (Cosmetic procedure was not related to Procedure).

**Table 4:** Top ten of LR classes according to the systematic naming metric, showing their hierarchy in SNOMED CT

LR class (label)	Hierarchy
411123000 (diagnostic allergen extract)	Pharmaceutical/biologic product
256259004 (pollen)	Substance
24851008 (deoxyribonucleic acid)	Substance
263490005 (status)	SNOMED CT model component
257351008 (shunt)	Physical object
87612001 (blood)	Substance
449872003 (powder)	Substance
264193005 (segment)	Qualifier value
255711007 (pattern)	SNOMED CT model component
277536004 (serogroup)	Qualifier value

#### Systematic naming metric

The systematic naming metric accounts for the ratio of taxonomically related classes, which share lexical regularities. As in the case of the LSLD metric, only the LR associated with the LR classes was considered. It was calculated as described in Algorithm 2. First, we obtained all the LRs appearing in the class labels of the input ontology and the corresponding LR classes. Then, for each LR class, we obtained all of its subclasses. Finally, for each subclass, we checked whether it was a positive case (whether it exhibited the label of the LR class) or a negative case (whether it did not exhibit the label of the LR class). Thus, the ratio of positive/negative cases could be obtained.

**Table 5:** Bottom ten of LR classes according to the systematic naming metric, sorted by negative cases

LR class (label)	LR class depth	Positive cases (depth) (distance)	Negative cases (depth) (distance)	Metric value
385268001 (oral dose form)	4	0 (NA) (NA)	61 (5.607) (1.607)	0
273248003 (action)	6	0 (NA) (NA)	27 (7.259) (1.259)	0
385287007 (parenteral dose form)	4	0 (NA) (NA)	25 (5.16) (1.16)	0
740596000 (cutaneous dose form)	4	0 (NA) (NA)	24 (5.167) (1.167)	0
10546003 (site)	6	0 (NA) (NA)	11 (7.545) (1.545)	0
133936004 (adult)	5	0 (NA) (NA)	6 (6.5) (1.5)	0
260726005 (part)	7	0 (NA) (NA)	4 (8) (1)	0
246176004 (form)	7	0 (NA) (NA)	3 (8.667) (1.667)	0
738984000 (parenteral)	4	0 (NA) (NA)	3 (5) (1)	0
116154003 (patient)	5	0 (NA) (NA)	2 (6) (1)	0

In the example shown in Fig. 1, there were two LR classes, namely, Procedure and Administrative procedure. Procedure had three subclasses (Administrative procedure, Death administrative procedure, and Biopsy administration), so it had two positive cases (Administrative procedure and Death administrative procedure), and one negative case (Biopsy administration). In the case of the class Administrative procedure, it had two subclasses (Death administrative procedure and Biopsy administration). Thus, Death administrative procedure was a positive case and Biopsy administration, a negative one. Hence, the value of the metric was 3/5. 
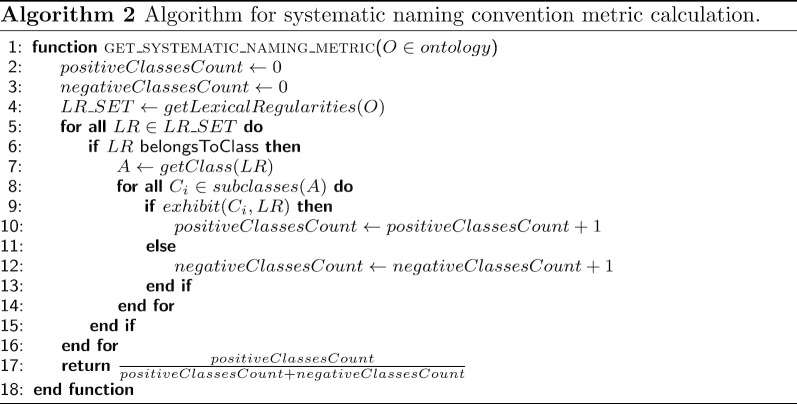


### Evaluation method

In this section, we describe the proposed application of the previous metrics to a given ontology with the purpose of contributing to its quality assurance. The first step is to obtain the values of the readability and structural accuracy metrics. Then, additional information can be captured for the structural accuracy metrics in order to support the analysis of the results. As mentioned earlier, both the LSLD and the systematic naming metrics are ratios accounting for the fulfilment of two of the best practices, and both use LR classes as the key elements. In our evaluation, we will also use the following information for each LR class:

1. Depth of the LR class in the hierarchy.
2. Number of positive cases.
3. Average taxonomic distance from the classes belonging to the positive cases set to the LR class. This taxonomic distance is the length of the shortest path between two classes by following is-a relationships.
4. Number of negative cases.
5. Average taxonomic distance from the classes belonging to the negative cases set to the LR class.
6. Value of the metric applied only to the LR class, that is, the ratio of positive cases to the total number of cases for this LR class.

We will then analyse the significance of the difference between the distance to the LR class of positive and negative cases by applying the Wilcoxon test [48] and whether there is a correlation between the depth of the LR classes in the ontology and their individual metric values by applying the Spearman correlation test [49]. This will be done for both LSLD and systematic naming.

**Table 6:** Bottom ten of LR classes according to the systematic naming metric, showing their hierarchy in SNOMED CT

LR class (label)	Hierarchy
385268001 (oral dose form)	Qualifier value
273248003 (action)	SNOMED CT model component
385287007 (parenteral dose form)	Qualifier value
740596000 (cutaneous dose form)	Qualifier value
10546003 (site)	SNOMED CT model component
133936004 (adult)	Social context
260726005 (part)	SNOMED CT model Component
246176004 (form)	SNOMED CT model Component
738984000 (parenteral)	Qualifier value
116154003 (patient)	Social context

## Results

We applied our framework to all of the available SNOMED CT versions from 2011 to 2019. The RF2 native SNOMED format was converted into OWL through the SNOMED CT OWL toolkit [50] and by applying the ELK reasoner [51]. The objective of the study was to provide an overview of the evolution of the readability and structural accuracy of SNOMED CT (see ‘Additional file 1’ in the GitHub repository for the complete results) and to detect the potential issues in the aspects analysed, thus contributing to its quality assurance. Moreover, we will carry out some studies on the most recent version available at the moment of writing this paper. The ‘Readability analysis’ section includes the results obtained after applying the readability metrics, whereas the ‘Structural accuracy’ section presents the results obtained for the structural accuracy metrics.

**Table 7:** Top ten of LR classes according to the LSLD metric, sorted by metric value first, and positive cases later

LR Class (label)	LR class depth	Positive cases (depth) (distance)	Negative cases (depth) (distance)	Metric value
736849007 (conventional release)	4	6071 (7.082) (3.082)	0 (NA) (NA)	1
385268001 (oral dose form)	4	2955 (5.725) (1.725)	0 (NA) (NA)	1
421026006 (conventional release oral tablet)	5	2286 (7.212) (2.212)	0 (NA) (NA)	1
385287007 (parenteral dose form)	4	1613 (5.581) (1.581)	0 (NA) (NA)	1
420692007 (conventional release oral capsule)	5	696 (7.129) (2.129)	0 (NA) (NA)	1
740596000 (cutaneous dose form)	4	566 (5.783) (1.783)	0 (NA) (NA)	1
736847009 (prolonged-release)	5	398 (6.96) (1.965)	0 (NA) (NA)	1
272673000 (bone structure)	7	372 (10.288) (3.293)	0 (NA) (NA)	1
282721001 (fluoroscopic guidance)	6	877 (6.523) (0.875)	1 (7) (1)	0.999
19830006 (blood group antibody)	9	715 (11.292) (2.365)	2 (5.5) (3.5)	0.997

**Table 8:** Top ten of LR classes according to the LSLD metric, showing their hierarchy in SNOMED CT

LR class (label)	Hierarchy
736849007 (conventional release)	Qualifier value
385268001 (oral dose form)	Qualifier value
421026006 (conventional release oral tablet)	Qualifier value
385287007 (parenteral dose form)	Qualifier value
420692007 (conventional release oral capsule)	Qualifier value
740596000 (cutaneous dose form)	Qualifier value
736847009 (prolonged-release)	Qualifier value
272673000 (bone structure)	Body structure
282721001 (fluoroscopic guidance)	Procedure
19830006 (blood group antibody)	Substance

### Readability analysis

Figure 2 shows that the readability metrics defined in the ‘Readability metrics’ section provided stable values for the different SNOMED CT versions processed. Regarding the classes, every class had at least two names: a qualified name and a preferred name, which were translated into rdfs:label and skos:prefLabel, respectively; nonetheless, there were classes with more than one preferred name, which led to the value of labels per class to be slightly higher than 2 for all of the SNOMED CT versions considered. With respect to the synonyms of the classes, the corresponding metric had values slightly higher than 0.5, which remained stable for the different versions. The lowest values for classes were obtained for the number of descriptions per class. In this case, the value of the metric was close to 0, indicating that only very few classes included descriptions. Regarding object properties, they usually had a qualified name and a preferred name, which led to a value close to 2 for such a metric; this value was slightly lower in the versions published before 2013-07-31, reaching a value of exactly 2 after this date. The number of synonyms per object property was stable for the different versions, with a value of around 0.1, which was lower than the number of synonyms per class. The number of descriptions per object property was the most varying metric over time. The object properties were not annotated with descriptions until 2015. In this year, the value reached 0.25. From 2015 to 2019, the value of this metric gradually increased to 0.37. Finally, on the one hand, the SNOMED CT ontology did not contain datatype properties, and, on the other hand, the annotation properties used in the ontologies did not have metadata, which led to a zero value for all of the metrics; thus, both annotation and data type properties are not seen in Fig. 2.

**Table 9:** Bottom ten of LR classes according to the LSLD metric, sorted by negative cases later

LR Class (label)	LR class depth	Positive cases (depth) (distance)	Negative cases (depth) (distance)	Metric value
42504009 (containing)	6	0 (NA) (NA)	20803 (6.678) (0.992)	0
255503000 (entire)	6	0 (NA) (NA)	14678 (10.842) (4.844)	0
18720000 (in)	6	0 (NA) (NA)	12671 (6.699) (1.228)	0
20401003 (with)	6	0 (NA) (NA)	10616 (7.495) (1.769)	0
260548002 (oral)	7	1 (6) (1)	7531 (6.59) (0.713)	0
246176004 (form)	7	0 (NA) (NA)	6519 (5.737) (1.351)	0
255333006 (conventional)	5	0 (NA) (NA)	6086 (7.082) (2.083)	0
86495002 (for)	6	0 (NA) (NA)	5715 (6.95) (1.501)	0
420862001 (on)	5	0 (NA) (NA)	4821 (6.873) (1.91)	0
733021006 (system)	5	0 (NA) (NA)	4068 (6.481) (1.545)	0

**Table 10:** Bottom ten of LR classes according to the LSLD metric, showing their hierarchy in SNOMED CT

LR Class (label)	Hierarchy
42504009 (containing)	Qualifier value
255503000 (entire)	Qualifier value
18720000 (in)	SNOMED CT model component
20401003 (with)	SNOMED CT model component
260548002 (oral)	Qualifier value
246176004 (form)	SNOMED CT model component
255333006 (conventional)	Qualifier value
86495002 (for)	Qualifier value
420862001 (on)	Qualifier value
733021006 (system)	Qualifier value

**Table 11:** Number of LR classes per hierarchy, sorted by number of LR classes

Hierarchy	Number of LR classes
Qualifier value	177
SNOMED CT model component	43
Substance	39
Body structure	34
Clinical finding	32
Procedure	22
Observable entity	7
Physical object	5
Social context	5
Environment or geographical location	4
Organism	2
Pharmaceutical/biologic product	2
Event	1
Physical force	1
Record artifact	1
Special concept	1
Specimen	1
Staging and scales	1
Situation with explicit context	0

Next, we will summarise the results on the latest SNOMED CT version. The complete data can be found in ‘Additional file 2’ in the GitHub repository. With respect to classes (see Fig. 3), the ontology contained a total of 350,711 classes. There were 23,513 (6.7%) classes annotated with three labels, and 327,198 (93.3%) with two labels, which led to an average of 2.07 names per class; in the case of synonyms, each class had a mean of 0.53 synonyms, having a median of 0. There were 13,068 (3.73%) with more than two synonyms, the class with ID 37810007 (myeloid leukaemia) being the most enriched one in this sense, with 33 synonyms. 25,957 (7.4%) classes showed exactly two synonyms, whereas 82,009 (23.39%) had a unique synonym. In contrast, there were 229,677 (65.49%) classes without synonyms. When we inspected the descriptions, we found 4062 (1.16%) classes annotated with one description and 1027 (0.29%) including two descriptions; however, there were 345,622 (98.55%) classes without a description.

The ontology contained 120 object properties (see Fig. 3), and all of them had two names. There were 114 (95%) object properties without synonyms; 5 (4.17%) with one synonym; and only the object property with ID 288556008 (before) had two synonyms. There were 78 (65%) object properties without description; 39 (32.5%) object properties had one description; and 3 (2.5%) object properties had two descriptions.

**Table 12:** Systematic naming metric values for each SNOMED CT hierarchy, including the number of LR classes in the hierarchy, and the counts of both positive and negative cases, sorted by the metric value

Hierarchy	LR classes	Positive cases	Negative cases	Metric value
Record artifact	1	3	0	1.00
Event	1	75	2	0.97
Pharmaceutical/biologic product	2	730	22	0.97
SNOMED CT model component	43	217	88	0.71
Organism	2	1735	1042	0.62
Specimen	1	1024	676	0.60
Qualifier value	177	870	719	0.55
Environment or geographical location	4	143	133	0.52
Physical force	1	32	38	0.46
Observable entity	7	1443	1939	0.43
Substance	39	8496	39131	0.18
Procedure	22	15463	79584	0.16
Clinical finding	32	16556	90841	0.15
Body structure	34	1680	13527	0.11
Physical object	5	1452	13981	0.09
Social context	5	25	296	0.08
Special concept	1	0	0	
Staging and scales	1	0	0

**Table 13:** LSLD metric values for each SNOMED CT hierarchy, including the number of LR classes in the hierarchy, and the counts of both positive and negative cases, sorted by the metric value

Hierarchy	LR classes	Positive cases	Negative cases	Metric value
Pharmaceutical/biologic product	2	730	62	0.92
Event	1	475	56	0.89
Specimen	1	1462	389	0.79
Body structure	34	21979	6975	0.76
Organism	2	2203	1096	0.67
Clinical finding	32	17069	10199	0.63
Procedure	22	16322	11050	0.60
Physical force	1	254	215	0.54
Physical object	5	2287	2776	0.45
Substance	39	15404	22749	0.40
Observable entity	7	1902	2960	0.39
Qualifier value	177	58589	180390	0.25
Environment or geographical location	4	143	1650	0.08
Social context	5	87	6898	0.01
Record artifact	1	3	410	0.01
SNOMED CT model component	43	217	69645	0.00
Special concept	1	0	452	0.00
Staging and scales	1	0	354	0.00

### Structural accuracy

Figure 4 shows the values obtained for the systematic naming and the LSLD metrics. Both metrics remained stable for the different SNOMED CT versions. The values of the LSLD metric were between 0.27 and 0.3, which decreased from 2016-07-31 to 2018-01-31, and increased since then, reaching its maximum value for the most recent version. The values of the systematic naming metric were around 0.17, with a minimum of 0.169 and a maximum of 0.18.

When we focused on the SNOMED CT version of 2019-07-31, we obtained a value of 0.171 for the systematic naming metric, and 0.304 for the LSLD metric. 378 LR classes were identified in this version, including domain concepts, such as ‘Hemoglobin’, ‘Injection’, or ‘Neoplasm’; and more general concepts such as ‘Right’, ‘Left’, ‘On’, ‘Does’, or even numbers. Moreover, we found 43 cases in which a label was shared between two classes. Some examples of this were the lexical regularity ‘Open wound’, which was the label of the SNOMED CT concepts 125643001—Open wound (disorder)—and 59091005—Open wound (morphologic abnormality)—; and the lexical regularity ‘Substance’, which was the label of the concepts 261217004—Substance (attribute)—and 105590001—Substance (substance)—.

The systematic naming metric could not be evaluated for 160 LR classes, such as 300594005 (Resonance), 62105006 (Compound), or 263767004 (Genital), because they were leaves and thus did not have subclasses. The value of the metric was 1 for 61 LR classes, which implied that all of their subclasses exhibited the LR of the parent class. Some examples of such LR classes were 411123000 (Diagnostic allergen extract), 263490005 (Status), and 257351008 (shunt). Tables 3 and 4 show the top ten LR classes for this metric, sorted by positive cases, and the SNOMED CT hierarchy containing the LR classes, respectively. In contrast, the value of the metric was 0 for 18 LR classes, such as 385268001 (Oral dose form) or 273248003 (Action); thus, their subclasses did not exhibit the lexical regularity of the parent. Tables 5 and 6 show the bottom 10 of these LR classes, sorted by negative cases, and the SNOMED CT hierarchy in which the concerning LR classes were located, respectively. The complete data for this metric can be found in the corresponding file in ‘Additional file 2’ in the GitHub repository. Figure 5 shows the distribution of the metric values by taking the LR classes as individuals. In this case, the value per LR class was 0.78 in median and 0.64 in mean. The difference between these individual systematic naming metric values with respect to the global one (0.171) was obtained by using the LR classes with a large number of negative cases, which drastically decreased the global metric value.

In addition, we compared the average taxonomic distance from the positive and the negative cases to their corresponding LR class. The positive cases presented a distance of 1.698 and 1.282 on average and median, respectively. For its part, the distance of negative cases was 2.138 on average and 1.875 on median. The Wilcoxon test showed that the difference in the taxonomic distance between the positive and the negative cases was statistically significant, with *p* value = $2.935 \cdot 10^{-5}; \alpha = 0.05$. Finally, we studied the correlation between the depth of LR classes and their individual metric value. For this, we applied the Spearman correlation test, which found that the depth of LR classes was slightly correlated to their individual metric value in a direct proportion, with $\rho = 0.2186587$; *p* value = $0.001157; \alpha = 0.05$.

With respect to the LSLD metric, eight LR classes obtained a value of 1 for the metric because the LR class was linked to all of the classes exhibiting the LR. For example, all the classes exhibiting the lexical regularity ‘conventional release’ (a total of 6071 classes) were semantically related to the LR class 736849007 (Conventional release). Tables 7 and 8 show the top 10 LR classes sorted by the metric value and positive cases, and the hierarchy in which the LR classes were located. The value of the metric was 0 for 122 LR classes. For instance, the LR class with ID 42504009 (Containing) had 0 positive cases and the largest number of negative cases, with 20, 803 classes, such as ‘Peanut containing products’ or ‘Fluid containing peripheral blood stem cells’, exhibiting the lexical regularity without being semantically related to the LR class. Another example was the LR class ‘Oral’, which had 7531 classes exhibiting the regularity but were not semantically related to it, such as ‘Oral air flow’ or ‘Oral RAE endotracheal tube’. In this case, only the class 314808005 (Oral site descriptor) was semantically related to the LR class ‘Oral’. Tables 9 and 10 show the bottom 10 LR classes according to LSLD value, sorted by the number of negative cases, and the hierarchy containing each LR class, respectively. The complete data about this metric are available in the corresponding file in ‘Additional file 2’ in the GitHub repository. Figure 5 shows the metric value distribution according to LR classes. In this case, the median value of the metric for each LR class was 0.033, which was lower than the global metric value (0.304), whereas the average value was 0.325, which was slightly higher.

In the case of the LSLD metric, the hierarchical distance of the negative cases to the corresponding LR class was 2.118 on median and 2.404 on average; whereas the distance of positive cases was 1.69 on median and 1.92 on average, reaching statistical significance according to the Wilcoxon test (*p* value = $2.428 \cdot 10^{-8};\,\alpha = 0.05$). Moreover, the Spearman correlation test found an inverse correlation between the depth of the LR classes and their individual metric values ($\rho = -0.19$; *p* value = $1.756 \cdot 10^{-4}; \alpha = 0.05$).

### Analysis by hierarchies

The results shown for the top and bottom 10 LR classes revealed that some SNOMED CT hierarchies were highly represented in these lists. Next, we analysed the values of the metrics for the 19 SNOMED CT hierarchies on the latest version of SNOMED CT.

#### Readability

All of the hierarchies presented a median of two labels per class, whereas the average number was slightly higher because of the existence of outlier classes with three labels. The hierarchy with the highest average number of labels per class was ‘Special concept’ (2.15). The lowest value was reached for ‘Organism’ (2).

The number of descriptions per class was also homogeneously distributed along the different hierarchies. The median value of descriptions per class was 0 for all the hierarchies. The highest mean was obtained for ‘Clinical finding’ (0.04 descriptions per class).

The number of synonyms per class showed the largest variation among the hierarchies (see Fig. 6). All the hierarchies had a median of 0, except for ‘Organism’ and ‘Staging and scales’, whose median was 1. ‘Organism’ and ‘Staging and scales’ obtained the largest mean values (0.76 and 0.69, respectively), followed by ‘Substance’ (0.65) and ‘Clinical finding’ (0.6). In contrast, ‘Pharmaceutical/biologic product’ and ‘Event’ had the lowest values for the average number of synonyms per class (0.04 and 0.1, respectively).

#### Structural accuracy

The 378 detected LR classes were heterogeneously distributed along the SNOMED CT hierarchies. Table 11 shows the number of LR classes per hierarchy. The most represented hierarchy was ‘Qualifier value’ with 177 LR classes, followed by ‘SNOMED CT Model Component’ with 43 LR classes, and ‘Substance’, with 39 LR classes. In contrast, no LR classes were found in the ‘Situation with explicit context’ hierarchy.

We calculated the absolute value of the structural accuracy metrics for each SNOMED CT hierarchy. This value was computed by dividing the number of positive cases by the total number of cases per hierarchy. Table 12 shows the value of the systematic naming metric for each hierarchy, together with the number of LR classes and the count of positive and negative cases per hierarchy, and sorted by the metric value. ‘Record artifact’, ‘Event’, and ‘Pharmaceutical/biologic product’ have the highest values (over 0.97); however, these hierarchies only contain one or two LR classes. The fourth hierarchy in this ranking was ‘SNOMED CT Model Component’, which reached a value of 0.71 having 43 LR classes. Examples of other hierarchies containing a significant number of LR classes are ‘Qualifier value’ (0.55), ‘Substance’ (0.18), ‘Procedure’ (0.16), ‘Clinical finding’ (0.15), and ‘Body structure’ (0.11).

Figure 7 shows the box plots for the systematic naming metric per hierarchy. The hierarchies whose LR classes had a higher mean value for the systematic naming metric were ‘Record artifact’, ‘Event’, ‘Pharmaceutical/biologic product’, and ‘Organism’, but the number of LR classes associated with them was at most two. The fifth hierarchy with the highest value for the metric was ‘Substance’, whose 39 LR classes reached a mean value of 0.72. Other examples were ‘Body structure’, ‘Clinical finding’, ‘Procedure’, or ‘Qualifier value’ whose LR classes had a mean of 0.55, 0.75, 0.57, and 0.67, respectively.

Table 13 shows the values of the LSLD metric for each hierarchy. Similar to the systematic naming, the highest value was obtained for hierarchies with at most two LR classes: ‘Pharmaceutical/biologic product’ (0.92), ‘Event’ (0.89), and ‘Specimen’ (0.78). The values provided by hierarchies with a higher number of LR classes varied from ‘Body structure’ (0.76) to ‘Qualifier value’ (0.24).

Figure 7 shows the individual analysis performed for the LR classes, grouping them by hierarchy. The highest mean value for the LSLD metric was observed for hierarchies with less than two LRs, namely ‘Pharmaceutical/biologic product’ (0.92), ‘Event’ (0.9), ‘Specimen’ (0.79), and ‘Organism’ (0.76). Amongst the hierarchies with more than two LRs, ‘Procedure’ had 22 LR classes and a mean value of 0.73; ‘Clinical finding’ had 32 LRs and a mean value of 0.56; ‘Substance’ had 39 LR classes and a mean value of 0.41; and ‘Qualifier value’ had 177 LR classes and a mean value of 0.22.

## Discussion

In this paper, we have defined a set of metrics for measuring the readability and the structural accuracy of ontologies. Our method allows for identifying which parts of the ontology have low readability or structural accuracy. The metrics provide information about the engineering of the ontology that permit one to detect potential flaws and errors and to evaluate from the metrics’ perspective the effect of modelling decisions; all of these aspects are relevant to the quality assurance purpose. In general, low values of the metrics or negative cases in the structural accuracy analysis are expected to be related to the flaws in the engineering of the ontology, but they may also be the consequence of the desired modelling decisions. In this work, the metrics were applied to different versions of SNOMED CT and, in general, all of them showed values that remained stable in time (see Figs. 2 and 4). This stability of the metrics could be interpreted as a maturity indicator of this ontology, implying that the same principles were applied in SNOMED CT modelling with time.

### Readability

In this work, readability was approached as the existence of human readable descriptions in the ontology, such as comments, labels, or captions [20]. As mentioned in the ‘State of the art’ section, readability is usually measured through the annotation properties. There exist basic metrics, such as those implemented in Protégé [36], that count the number of annotation properties and the number of annotation assertion axioms. More complex metrics take into account the meaning of the annotation properties in order to select those that refer to readability. For example OntoQA [20] uses rdfs:label and rdfs:comment annotation properties. Nonetheless, this may not be sufficient because there are emerging vocabularies that are widely used by the semantic web community for including elements such as labels, synonyms, or descriptions in ontology entities. In order to adapt to the community practice, we analysed the use of annotation properties in BioPortal ontologies. We found that four annotation properties referring to labels, seven referring to synonyms, and five referring to descriptions had sufficient use to take them into account when analysing readability. These properties were provided by the following resources: Simple Knowledge Organization System (skos) [37], Resource Description Framework Schema (rdfs) [52], schema.org [53], oboInOwl [54], Information Artifact Ontology (IAO) [55], Dublin Core Terms (dcterms) [38], and National Cancer Institute Thesaurus (NCIT) [56].

We applied this result to the study of readability in SNOMED CT. For the conversion to OWL, we used skos:prefName and rdfs:label for labels, skos:altName for synonyms, and skos:definition for descriptions. The results obtained for the readability metrics showed that the values remained stable in time, except for the descriptions per object property, which was improved in January 2015 by including descriptions in these elements (see Fig. 2).

The number of descriptions was small in the most recent version included in the study, being close to 0 for descriptions per class and 0.37 for descriptions per object property. Ideally, each ontology entity should have at least one human-readable description, which helps it to be understandable by human beings. In contrast, the number of names per class and per object property was around 2, and this value was stable along different hierarchies, which was a rich value for the metric. Finally, we found that there existed 0.53 synonyms per class and 65.49% of the classes did not contain any synonym. Our analysis per hierarchy might help to detect which hierarchies had the least number of synonyms (see Fig. 6). The values of the metrics for object properties were lower, and 95% of them did not have any synonym. This could be improved by adding synonyms to these classes and properties. These metrics can be applied to any ontology to detect which type of ontology entity (class or object property) should be enriched with a concrete type of annotation (labels, synonym or description). Our results also revealed that these readability metrics could be used for detecting potential changes in modelling decisions and, in case such changes are intended by the developers, the metrics provide information about the effects of the implementation of such decisions. Consequently, they are useful for the ontology quality assurance process.

### Structural accuracy

The structural accuracy metrics analyse the consistency between the lexical content included in the labels and the logical one expressed in the axioms by following the lexically suggest logically define principle [40]. These aspects are usually modelled by calculating different hierarchical-based features, where higher values of depth and breadth variance in the class hierarchy tree are associated with better semantic relations [19, 39]. Our solution is in line with [44, 45], which proposed a lexical study in order to discover the possible inconsistencies. [44] focused on the semantics of sets that group lexically similar concepts, and [45] used subgraphs enriched with lexical characteristics obtained along the is-a relationships. The approach followed in our work was to focus on the LR classes, which are classes whose label is a lexical regularity. We considered that LR classes provide the relevant domain knowledge and domain concepts that are reused for creating other classes in the ontology. Two metrics have been proposed based on such LR classes: (1) LSLD, which accounts for the existence of semantic links between LR classes and classes to which they are lexically connected; and (2) systematic naming, which focuses on the degree to which the subclasses of the LR classes contain the corresponding LR. The LSLD metric facilitates the detection of situations of potentially incorrect or suboptimal axiomatisation of the ontology, and the systematic naming metric facilitates the detection of the possible inconsistencies in the naming of the entities or in the ontology hierarchy. All of these aspects are relevant in ontology quality assurance processes.

With respect to the structural accuracy of SNOMED CT, the systematic naming metric had a general value closer to 0.17 for all of the SNOMED CT versions. We found that the use of synonyms resulted in lower values for the metric. We show next some examples of negative cases found by the metric for the latest version. The complete list is available in ‘Additional file 4’ in the GitHub repository. An example is the class 75478009 (Poisoning), which has subclasses such as 197359004 (Toxic liver disease with chronic persistent hepatitis) or 111776002 (Toxic effect of hydrocarbon gas). These subclasses do not exhibit the LR ‘poisoning’; however, they use the word ‘toxic’, which could be a synonym of ‘poisoning’. Another example is the LR class 34896006 (Incision), which has subclasses such as 150062003 (Osteotomy) or 359696001 (Colpotomy for pelvic peritoneal drainage), being ‘osteotomy’ and ‘colpotomy’ hyponyms of ‘incision’. In these cases, replacing the corresponding term(s) in the negative case would fix these situations. For example, the LR class 782161000 (Bite) has 75 subclasses that exhibit the lexical regularity ‘bite’; however, there are two subclasses in which the LR is not exhibited, namely the class 242599009 (Stung by cone shell), and the class 262551003 (Flea bites), which could be renamed as ‘bite by cone shell’ and ‘bite by flea’, respectively, in order to increase the value of the metric. Additionally, we found that the LR classes located in the upper positions in the hierarchy were likely to obtain lower values for the systematic naming metric and that negative cases were further from the LR class than the positive ones (see the ‘Structural accuracy’ section). This implied that the naming convention was likely to be broken for the most specific classes (higher depth) in the ontology. These aspects are exemplified in the LR class 64572001 (Disease), which is a direct subclass of ‘clinical finding’. In this case, the LR class has 1956 positive cases and 74,026 negative cases, thus obtaining a low value of 0.026 for the metric. This low value could be explained because higher LR classes denote general concepts and their lexical regularities are difficult to maintain in the labels of their subclasses, as they became increasingly specific and complex with an increase in the hierarchy depth. In fact, the positive cases had an average distance of 4.340 to the Disease class, whereas the average distance of the negative cases was 4.680. In this case, the low value of the metric was justified by the nature of the concept ‘clinical finding’, as it would not be usual to name subclasses such as Disease as ‘Clinical finding diseases’ or any other combination including ‘clinical finding’. This implied that the value of this metric was to provide information about the engineering of the ontology, but the value had to be analysed and interpreted by the ontology developers, as low values could be justified by the nature of the domain knowledge.

The analysis of the systematic naming metric by SNOMED CT hierarchy could help ontologists to analyse the results by module, which could be maintained and developed by different people and even require different modelling patterns. For example, the hierarchy ‘body structure’ had an absolute metric value of 0.11 (see Table 12); however, the average individual values of the LR classes belonging to this hierarchy was 0.55 (see Fig. 7). This implied that there existed LR classes in this hierarchy with many more negative cases than positive ones, which had a negative impact on the value of the metric. Some examples are 419351001 (Sinus), with 41 positive and 875 negative cases; or 2483006 (Cavity), with 9 positive and 726 negative cases.

The LSLD metric showed values closer to 0.3 for the different SNOMED CT versions. In this case, we also performed a further study of the latest version based on the negative cases provided by this metric (see ‘Additional file 3’ in the GitHub repository for a complete list of negative cases). We found that a considerable number of negative cases were a result of very general classes. For example, the label of the LR class 38112003 was ‘1’, and this LR appeared in 1324 classes, from which only 386 were semantically related to the LR class, thus providing an LSLD metric value of 0.29. We considered a similar scenario for other LR classes that were numbers, including 19338005 (2), 79605009 (3), and 3445001 (10), among others. It could be argued that if the classes exhibited LR, there were numbers that were linked to these LR classes. Other examples of general LR classes with a high number of negative cases are 42504009 (Containing), 255503000 (Entire), and 18720000 (In), as they are very common words that appear in many class labels, although they are not used for describing these classes semantically. Prepositions such as ‘In’ are sometimes considered stopwords and therefore excluded from previous studies. In our case, we preferred to consider them in this study.

Next, we focused on the existence of object properties with the LR classes. For example, the LR class 269736006 (poisoning of undetermined intent) was semantically related to the 501 classes that exhibited the lexical regularity, but there were 2 negative cases. These negative cases were the classes 291421002 (Alternative medicine poisoning of undetermined intent) and 242976002 (Poisoning of undetermined intent by non-drug substance), which had zero object properties associated. The object properties might be related, for instance, to laterality, which are important in SNOMED CT. An example is the class 7771000 (Left), which is related to 287045000 (Pain in left arm).

The analysis of the LSLD metric by the SNOMED CT hierarchy could also help to identify which hierarchies follow better the lexically suggest, logically define principle. An example is the ‘Qualifier value’ hierarchy, which has an LSLD metric value of 0.25, having 58,589 positive and 180,390 negative cases derived from the 177 LR classes detected in the hierarchy (see Table 13). Moreover, the mean and the median of the individual metrics per LR class was 0.22 and 0, respectively. Further, we detected that most of the very general LR classes, such as 255503000 (Entire), 42504009 (Containing), and 86495002 (for), belong to this hierarchy (see Table 10), resulting in a low value for the metric. In addition, we detected LR classes such as 255551008 (posterior) or 303231004 (intracranial) with 1853 and 615 negative cases, respectively, which are not being used for defining classes such as 256707009 (Posterior cervical flap), 202889007 (Posterior shin splints), 244951004 (Entire posterior muscle of abdomen), 44823006 (Intracranial embolic abscess), and 128319008 (Intracranial structure). Again, the LSLD metric provides information about the engineering of the ontology. The results must be interpreted by the domain experts. In case, they agree with the shortcomings spotted by the metric, they will have to make decisions about the content of the ontology. Consequently, the metric is useful for the quality assurance of the ontology.

In this study, we focused on SNOMED CT, but the metrics could be applied to other ontologies. Note that despite the fact that we focused on the values of the metrics, our method could retrieve the negative cases, whose revision should be part of the quality assurance of the ontology. The study of negative cases could help to determine the correctness of the position of a class in the hierarchy, to suggest the renaming of the class to be in concordance with the name of its ancestors, or to suggest that there are missing object properties between concepts.

### Limitations and future work

The metrics presented in this work permit the analysis of certain features of ontologies, but the global evaluation of the quality of an ontology requires one to combine them with other metrics in order to develop a complete quality assurance framework. In this sense, we plan to include the new metrics into our OQuaRE framework [15], which currently includes 19 ontology metrics. The integration of the new metrics will require us to map the range of values of the metrics to the quality scores 1 to 5, which in turn requires a study of different ontologies and repositories. Hence, we will perform a systematic analysis of the content of repositories such as the OBO Foundry [57], BioPortal [47], and UMLS [58] with the purpose of characterising the current biomedical ontologies from the perspective of the metrics, hence enabling a comparative analysis of the metrics and ontologies. As future work, we also plan to improve the detection of LRs and the detection of negative cases in systematic naming and LSLD metrics, and to apply the novel metrics to a different corpus of ontologies. We believe that the lemmatisation or normalisation of the labels would contribute to the improvement of the detection of LRs. Regarding the negative cases, our lexical analysis has the limitation of not taking into account the cases in which a lexical regularity is exhibited without doing an exact match, thus providing false negatives. This is exemplified by the LR class 269736006 (Poisoning of undetermined intent), which has subclasses such as 291368008 (Poisoning caused by herbal asthma mixture of undetermined intent) and 291990000 (Poisoning caused by angiotensin-converting enzyme inhibitor of undetermined intent). In this case, both the subclasses do not provide an exact match with the regularity ‘Poisoning of undetermined intent’; however, we can consider that they are exhibiting it by including extra text in the middle. Another improvement is to filter the negative cases for providing useful information about how to improve the ontology. In the case of SNOMED CT, we can ignore classes in the upper levels, as they are likely to present many negative cases that cannot been fixed because of the complexity of the ontology itself. Nonetheless, this is a difficult task, because not all of the ontologies present the same organisation. We also plan to enable filters to exclude certain parts of the ontology from the analysis.

## Conclusion

The quality assurance of biological and biomedical ontologies is critical for the success of their application to support research. In this paper, we addressed two relevant features of biomedical ontologies, namely readability and structural accuracy. Readability not only improves the understanding of ontologies by humans but also empowers their use in natural language processing tasks, such as named entity recognition. An accurate structure of the ontology guarantees that ontologies are built by applying the best practices and methodologies that make their development and maintenance easier. Our metrics were applied to SNOMED CT, showing their capability to provide useful insights about the engineering of the ontology. Therefore, this work makes the following contributions: (1) a set of readability metrics based on the most frequently used annotation properties, (2) the use of lexical regularities to define two metrics related to structural accuracy, and (3) the generation of quality assurance information for SNOMED CT.

## Data Availability

The datasets generated and/or analysed during the current study are available in the GitHub repository https://github.com/fanavarro/lexical-analysis-snomed-ct.
